# Valproate associated brain volume‐loss in pediatric epilepsy—A case series

**DOI:** 10.1002/epi4.12807

**Published:** 2023-08-22

**Authors:** Johanna Umlauf, Alexander Rau, Theo Demerath, Thomas Bast, Jan Schönberger, Horst Urbach, Julia Jacobs, Kerstin A. Klotz

**Affiliations:** ^1^ Department of Neuropediatrics and Muscle Disorders, Center for Pediatrics, Medical Center–University of Freiburg, Faculty of Medicine University of Freiburg Freiburg Germany; ^2^ Department of Neuroradiology, Medical Center–University of Freiburg, Faculty of Medicine University of Freiburg Freiburg Germany; ^3^ Department of Diagnostic and Interventional Radiology, Medical Center–University of Freiburg, Faculty of Medicine University of Freiburg Freiburg Germany; ^4^ Epilepsy Center Kork Kehl Germany; ^5^ Freiburg Epilepsy Center, Medical Center–University of Freiburg, Faculty of Medicine University of Freiburg Freiburg Germany; ^6^ Alberta Children's Hospital, Alberta Children's Research Institute, Hodgekiss Brain Institute, Section of Pediatric Neurology University of Calgary Alberta Canada

**Keywords:** brain atrophy, case series, epilepsy, pseudoatrophy, valproate

## Abstract

Brain atrophy associated with valproate therapy is known from single case reports and is frequently accompanied by cognitive deterioration. We present a case series of incidental findings of brain volume loss in children treated with valproate and employed automatic brain volumetry to assess the effect size of volume loss. 3D T1w datasets were automatically segmented into white matter, gray matter, and cerebrospinal fluid using the SPM‐12 algorithm. Respective volumes of cerebrum and cerebellum were read out and normalized to the total intracranial volume. We identified six patients (median age 148.5 [85–178] months) who had received valproate for a median time of 5 (2–23) months prior to MRI in which a loss of brain volume was noted. None had reported the occurrence of new clinical symptoms. Volumetry showed a volume loss of up to 28% for cerebral GM, 25% for cerebellar GM, 10% for cerebral WM, and 20% for cerebellar WM. A volume loss of >5% in at least one of the subvolumes was found in all patients, with the more prominent volume loss in the cerebrum and in gray matter. In one patient, post‐valproate MRI was available and showed normalization of brain volume. Our case series indicates that valproate therapy might be associated with an asymptomatic volume loss of brain parenchyma in children with epilepsy and that this volume loss is assessable with automatic volumetry.

## 1 INTRODUCTION

Valproate (VPA) is a frequently prescribed, first‐generation antiseizure medication for treatment of both generalized and focal epilepsies. It is associated with different types of adverse drug reactions (ADR) ranging from drowsiness, changes in appetite and hematological changes to more severe ADR including pancreatitis, hepatotoxicity up to liver failure, and hyperammonemic encephalopathy with and without associated liver failure.^1^ The first case report of VPA‐associated brain atrophy in children with epilepsy was published over 30 years ago.^2^ Since then, seven case report of brain atrophy in children with epilepsy receiving VPA have been published.^3^, ^4^, ^5^, ^6^, ^7^, ^8^ Authors report the acute or subacute occurrence of symptoms, mainly somnolence and acute cognitive regression, but also tremor, nystagmus, and newly occurred challenging behavior leading to MRI in which cerebral and/or cerebellar atrophy was identified visually. Duration of VPA therapy before onset of symptoms varied between 3 weeks and >5 years. VPA serum levels were within the therapeutic range in all but one case and ammonia levels were only slightly elevated in two cases, normal in the others. Cognitive impairment was reported in all but one case, but formal neurocognitive testing was performed in a limited number of cases only. Most of the case reports indicate that the brain atrophy was reversible after discontinuation of VPA. In one case, genetic testing revealed a previously unknown mitochondrial mutation in *MTAPT8* gene.^7^ In all other cases, the pathophysiology of brain atrophy remained unknown. Two recent, retrospective studies in adults with epilepsy have shown lower volume of the whole brain and different subvolumes in patients treated with VPA. These studies thus suggest that VPA‐associated brain atrophy occurs asymptomatically and underreporting must be suspected.^9^, ^10^

This case series aimed to describe children with epilepsy in which a VPA‐associated brain volume loss was found in routine MRI and to assess the effect size of volume loss in cerebrum and cerebellum white matter (WM), gray matter (GM) and cerebrospinal fluid (CSF).

## 2 METHODS

We retrospectively identified children (0–18 years) with VPA treatment between 2011 and 2021 in which a loss of brain volume was reported in MRI and no obvious reasons for this were present (e.g., neurodegenerative disorders, storage diseases, Rasmussen Encephalitis, Sturge–Weber syndrome, bullemia/anorexia nervosa, hemimegalencephaly, history of cerebral radiation therapy, history of steroid or phenytoin treatment within 6 months prior to MRI). Criteria for epilepsy were fulfilled according to International League Against Epilepsy (ILAE).^11^ MRI with 3D T1w‐dataset had to be available prior to VPA therapy and during VPA therapy. All MRI had to be acquired following a standardized protocol for evaluation of epilepsy patients, MRI with corresponding field strengths were selected whenever possible. Clinical information was collected from local health care systems. This study was approved by the institutional review board (Ethics Committee—University of Freiburg—20‐1190). The requirement for informed written consent was waived.

Data processing was implemented on a local instance of the postprocessing platform NORA (www.nora‐imaging.org). Using Matlab, 3D T1w datasets were automatically segmented into WM, GM, and CSF based on the tissue‐probability value using the SPM‐12 algorithm (The Welcome Centre for Human Neuroimaging, UCL Queen Square Institute of Neurology), followed by a case‐wise visual control of correct segmentation. Volumes of cerebrum and cerebellum were extracted in mm^3^, each subdivided into the subvolumes of WM, GM, and CSF (the latter for plausibility check). For this, masks of the cerebrum and cerebellum were generated in standard MNI space and warped to the individual space. All individual raw values were normalized to the total intracranial volume (TIV) of the respective patient (raw value subvolume [mm^3^]/TIV[mm^3^] = normalized subvolume).

## 3 RESULTS

We identified six patients (median age 148.5 [85–178] months) with genetic (*n* = 2), structural (*n* = 2), or unclassified (*n* = 2) epilepsy, who had received VPA for a median time of 5 months (range 2–49) prior to MRI in which volume‐loss was noted. Field strength of the corresponding MRI was equivalent in all patients besides patient #3 and #5 (first MRI 1.5T, second 3T). Detailed patients' characteristics are displayed in Table 1. In none of the patients, the occurrence of new clinical symptoms had been documented.

**TABLE 1:** Patients' characteristics.

ID	Sex	Epilepsy type or etiology	Comorbidities	ASM at First MRI Second MRI (Third) MRI	Age [m] at start of VPA	Age [m] at first atrophy	VPA dosage [mg/kg/day]^a^	VPA level [mg/L]^a^
1	M	Genetic (*NTRK* mutation), LGS	Autism	TPM VPA	84	133	23	113
2	M	Multifocal, etiology unknown	DM I	None VPA + LEV	94	98	10	81
3	F	Subependymal band heterotopia	None	CLB + STM VPA	159	164	27	51
4	F	Structural, DNET	None	OXC OXC + VPA OXC	176	178	17	81
5	M	Juvenile absence epilepsy	GMS	LTG VPA	162	175	61	131
6	M	Multifocal, etiology unknown	Autism	LCM + LEV+LTG VPA + LTG	80	85	9	Not available

^a^ At MRI with atrophy.

Abbreviations: CLB, clobazam; DM, diabetes mellitus; DNET, Dysembryoplastic neuroepithelial tumor; F, female; GMS, Gilbert‐Meulenkracht syndrome; LEV, levetiracetam; LTG, lamotrigine; M, male; m, months; OXC, oxcarbazepine; STM, sulthiame; TPM, topiramate; VPA, valproate.

**FIGURE 1:**
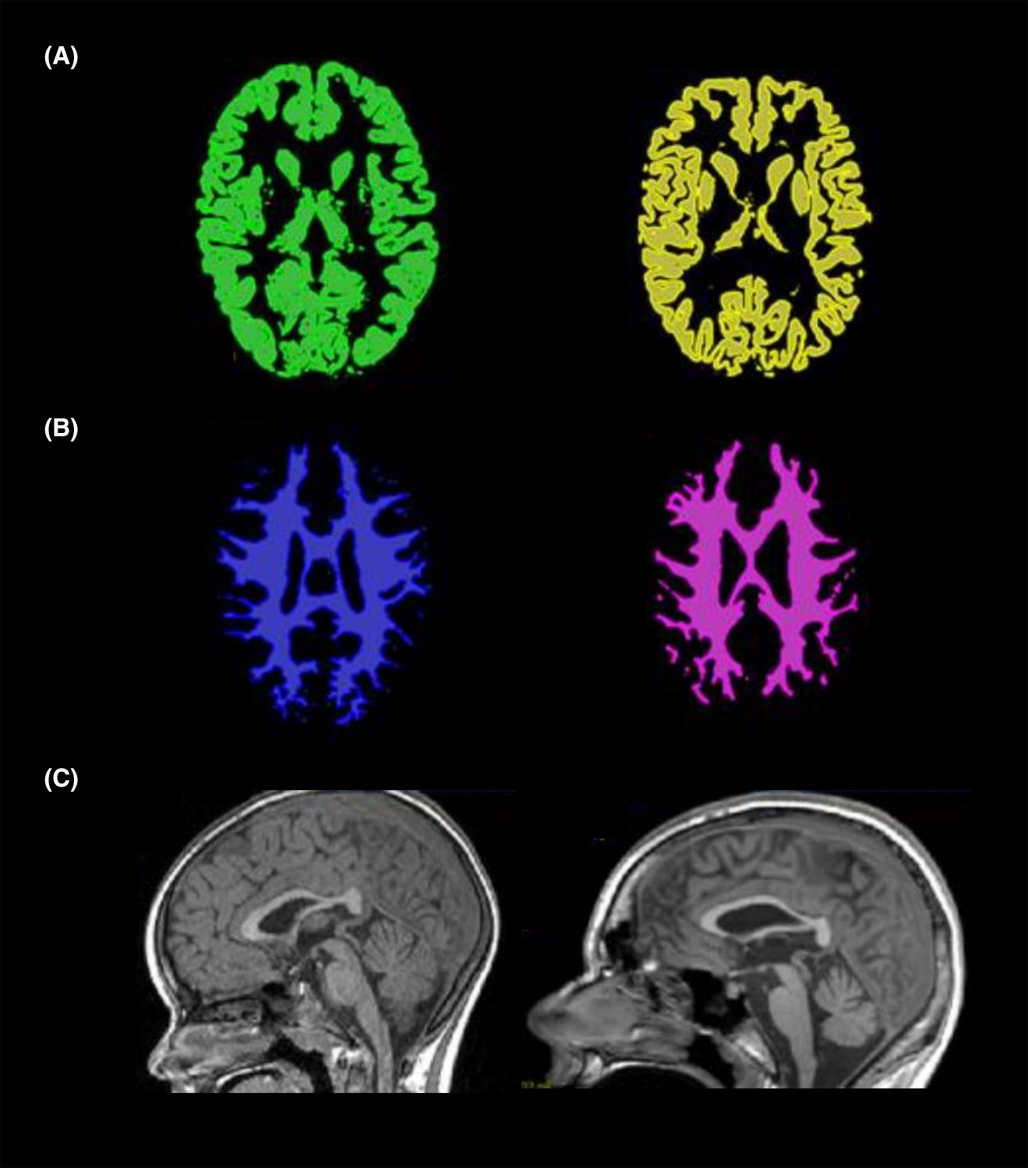
MRI of patient #1 at age 6.8 years prior to VPA initiation (left column) and at age 11.1 years on VPA treatment for 23 months (right column). Loss of cerebral gray matter (A), white matter (B) and cerebellar atrophy (C) are seen on second MRI compared to first one.

Supratentorial (cerebral) volumetry showed a volume loss of up to 28% for GM (median −10.4, range −7.3 to −28.4) and up to 10% for WM (median −9.5, range +5.1 to −10.4) with a gain of supratentorial CSF volume up to 132% (median 28.6, range +11.2 to +132.1). Infratentorial (cerebellar) volumetry showed a volume loss of up to 25% for GM (median −6.4, range −1.1 to −25.5) and up to 20% for WM (median −3.8, range +6.7 to −20.6) with a gain of infratentorial CSF volume up to 122% (median +49.8, range +13.1 to +122.5). Overall, a volume loss >5% was seen in 6/6 patients for cerebral GM, 2/6 for cerebral WM, 3/6 for cerebellar, GM and 3/6 for cerebellar WM. Figure 1 shows the MRI of patient 1 (male 6.8 years old) 2 months prior to and 4 years after initiation of VPA treatment. In one patient (female; 14.7 years old at VPA initiation), a follow‐up MRI was available for volumetry after discontinuation of VPA. In this patient, volumes of cerebral and cerebellar GM and WM not only normalized after discontinuation of VPA but also showed growth in all subvolumes compared to pre‐VPA. Volumetry results are displayed in detail in Table 2.

**TABLE 2:** Volumes of cerebral and cerebellar gray‐ and white matter and CSF, normalized to total intracranial volume before, during, and after VPA.

ID		MRI pre‐VPA	MRI during VPA	MRI post‐VPA	Δ volume pre‐ to during‐VPA MRI [%]	Δ volume pre‐ to post‐VPA MRI [%]
1	Age (m)	82	133			
	duration VPA (m)	‐	23			
	C‐GM	0.511	0.389		−24.0	
	C‐WM	0.226	0.202		−10.4	
	C‐CSF	0.127	0.277		+11.9	
	CL‐GM	0.085	0.064		−25.5	
	CL‐WM	0.012	0.013		+5.1	
	CL‐CSF	0.013	0.029		+122.5	
2	Age (m)	32	98			
	duration VPA (m)	‐	4			
	C‐GM	0.486	0.450		−7.3	
	C‐WM	0.199	0.189		−5.0	
	C‐CSF	0.174	0.218		+25.4	
	CL‐GM	0.087	0.080		−6.5	
	CL‐WM	0.013	0.011		−8.6	
	CL‐CSF	0.016	0.025		+49.9	
3	Age (m)	33	164			
	duration VPA (m)	‐	5			
	C‐GM	0.555	0.397		−28.4	
	C‐WM	0.207	0.217		+5.1	
	C‐CSF	0.111	0.258		+132.1	
	CL‐GM	0.080	0.068		−15.1	
	CL‐WM	0.009	0.011		−20.6	
	CL‐CSF	0.013	0.023		+74.7	
4	Age (m)	172	178	185		
	Duration VPA (m)	‐	2	VPA disc. for 7 m		
	C‐GM	0.416	0.385	0.445	−7.5	+6.9
	C‐WM	0.225	0.231	0.246	+2.7	+9.2
	C‐CSF	0.222	0.247	0.174	+11.2	−21.8
	CL‐GM	0.077	0.073	0.082	−4.5	+6.3
	CL‐WM	0.014	0.015	0.015	+6.7	+8.3
	CL‐CSF	0.019	0.021	0.012	+13.1	−34.8
5	Age (m)	146	175			
	Duration VPA (m)	‐	13			
	C‐GM	0.474	0.413		−13.0	
	C‐WM	0.274	0.250		−8.7	
	C‐CSF	0.115	0.200		+73.4	
	CL‐GM	0.083	0.078		−6.3	
	CL‐WM	0.012	0.012		−6.7	
	CL‐CSF	0.014	0.022		+49.7	
6	Age (m	66	85			
	Duration VPA (m)	‐	5			
	C‐GM	0.481	0.444		−7.8	
	C‐WM	0.240	0.228		−4.9	
	C‐CSF	0.149	0.196		+31.8	
	CL‐GM	0.077	0.076		−1.1	
	CL‐WM	0.012	0.012		−0.93	
	CL‐CSF	0.016	0.019		+14.91	

Abbreviations: C, cerebrum; CL, cerebellum; CSF, cerebrospinal fluid; GM, gray matter; M, months; VPA, valproate; WM, white matter.

## 4 DISCUSSION

We report on a case series of six children with brain volume loss associated with VPA treatment without the occurrence of new clinical symptoms. Our results provide evidence on the feasibility of automated volumetry to capture brain volume loss in children with VPA therapy. In previous case reports, atrophy was visually identified, which mostly relies on the widening of sulci and subjective reader suspicion. This limits not only accuracy but also the possibility to compare different brain regions as well as WM and GM. If the authors described the atrophy in more detail, it was either a diffuse cerebral and/or a cerebellar atrophy.^3^, ^5^ In our case series, cerebral volume loss was more prominent than cerebellar volume loss in five of the six patients. We also saw a more pronounced volume loss of GM than WM in the cerebrum in all patients. Contrariwise, in the cerebellum, GM loss could be more, less, or equally prominent than WM loss. Two cross‐sectional studies have addressed the question of VPA‐associated brain atrophy by volumetry and measuring cortical thickness in adults with epilepsy, comparing patients with and without VPA therapy.^9^, ^10^ In both studies, the VPA group showed reduced WM volume and cortical thickness. Considerable regional differences with a focus on the posterior parts of the brain (parietal and occipital lobe) were reported in both studies.

Previously published single case reports describe the occurrence of brain atrophy in context of acute or subacute symptoms as sedation or neurocognitive regression. In none of the patients in our case series, the occurrence of new symptoms was documented. Unfortunately, data from a formal cognitive testing were not available in a reasonable timeframe for the MRI. However, as all patients were followed up by an experienced neuropediatrician, informal assessments of cognitive function (e.g. school performance) were documented in all cases without signs of cognitive decline. As this is a retrospective study, milder symptoms could of course have been missed. Nevertheless, our data as well as the two retrospective studies in adult patients with epilepsy indicate the possibility of asymptomatic brain volume loss, which might lead to an underreporting of VPA‐associated brain volume‐loss.^9^, ^10^ Overall, the incidence of VPA‐associated brain volume loss remains unclear to date. It is also unknown, in which timeframe after VPA initiation a loss of brain volume occurs and what the risk factors contribute to this. Time from initiation of VPA to first documented brain volume loss was variable in our study but since it was an incidental finding in all cases, we cannot draw any conclusion of the influence of therapy duration on the risk of brain volume‐loss. Also, the small cohort does not allow for any conclusions of VPA dose and serum level on the risk of brain volume loss.

Most of the casuistics indicate that the atrophy was reversible after discontinuation of VPA, hence the term “pseudoatrophy”.^2^ Only in one patient of our series, a post‐VPA MRI was available, where we could confirm not only the reversibility of the volume loss but also signs of physiological growth compared to baseline values. Nevertheless, it remains unclear up to date if even a reversible “pseudoatrophy” might have neurocognitive consequences in a developing brain. Interestingly, Tondelli et al.^10^ showed, that patients who were not on VPA medication at the time of the MRI but had been exposed to VPA in the past showed equivalent cortical thinning or ventricle enlargement to patients still on VPA. This observation challenges the idea of reversibility of VPA‐associated brain volume loss, at least in adult patients.

Several authors have discussed pathophysiological considerations.^5^, ^9^, ^10^ Because of the rapid changes of brain volume especially after discontinuation, the favored pathomechanism in this context is a loss of water content due to osmotic shifts, but data that actually assess some of the discussed mechanisms are not available.

The accuracy and reproducibility of brain volumetry depend on field strength and protocol changes.^12^ While cross‐sectional structural MRI studies show reasonably large case–control effect sizes in volume in the range of up to 5%–10%, within‐subject brain morphometry studies show smaller regional brain volume change in the range of up to 0.5% per year.^12^ We used only MRI acquired following a standardized protocol and field strengths were equivalent in 4/6 patients, but the results of this retrospective case series might be limited by the comparison of MRI recorded at different scanners in some of the patients. Nevertheless, we observed a volume loss of clearly more than 5% in all patients for cerebral GM, which is considered more than the expected variation resulting from the use of different scanners or physiological factors.

One major limitation of all case reports, including ours, is the missing control group. In a large cross‐sectional study widespread patterns of altered subcortical volume and reduced GM thickness was observed in adults with epilepsy in comparison to healthy controls.^13^ Data in children are more limited but overall, there are clear indications that epilepsy can result in brain volume loss.^14^ In addition, our results might be limited by a selection bias, as visual assessment of volume loss has been shown to be unreliable.

## 5 CONCLUSION

Our case series indicates that VPA therapy might be associated with an asymptomatic volume loss of brain parenchyma in children with epilepsy, which can be measured with automated volumetry. Larger, cross‐sectional studies with age‐matched control group of children with epilepsy but no VPA treatment are needed.

## CONFLICT OF INTEREST STATEMENT

None of the authors has any conflict of interest to disclose.

## ETHICS STATEMENT

We confirm that we have read the Journal's position on issues involved in ethical publication and affirm that this report is consistent with those guidelines.
